# Learning and predicting the unknown class using evidential deep learning

**DOI:** 10.1038/s41598-023-40649-w

**Published:** 2023-09-09

**Authors:** Akihito Nagahama

**Affiliations:** The University of Electro-Communications, 1-5-1 Chofugaoka, Chofu, Tokyo, 182-8585 Japan

**Keywords:** Computer science, Information technology

## Abstract

In practical deep-learning applications, such as medical image analysis, autonomous driving, and traffic simulation, the uncertainty of a classification model’s output is critical. Evidential deep learning (EDL) can output this uncertainty for the prediction; however, its accuracy depends on a user-defined threshold, and it cannot handle training data with unknown classes that are unexpectedly contaminated or deliberately mixed for better classification of unknown class. To address these limitations, I propose a classification method called modified-EDL that extends classical EDL such that it outputs a prediction, i.e. an input belongs to a collective unknown class along with a probability. Although other methods handle unknown classes by creating new unknown classes and attempting to learn each class efficiently, the proposed m-EDL outputs, in a natural way, the “uncertainty of the prediction” of classical EDL and uses the output as the probability of an unknown class. Although classical EDL can also classify both known and unknown classes, experiments on three datasets from different domains demonstrated that m-EDL outperformed EDL on known classes when there were instances of unknown classes. Moreover, extensive experiments under different conditions established that m-EDL can predict unknown classes even when the unknown classes in the training and test data have different properties. If unknown class data are to be mixed intentionally during training to increase the discrimination accuracy of unknown classes, it is necessary to mix such data that the characteristics of the mixed data are as close as possible to those of known class data. This ability extends the range of practical applications that can benefit from deep learning-based classification and prediction models.

## Introduction

Deep learning is used for prediction, classification, and modeling in various fields, and deep learning models^1–5^ have demonstrated remarkable achievements in areas, such as medicine^6,7^, autonomous driving^8,9^, and stock market prediction^10^. There is uncertainty in the output of a deep learning method, particularly a prediction model. Thus, there are situations where the uncertainty needs to be output; for instance, when dataset shifts occur^11^ in medical image analysis^12,13^ and autonomous driving^14^ tasks.

Prediction models that output uncertainty or, in other words, have an output that means “I do not know,” include models based on Bayesian neural networks^15,16^ and Gaussian process models^17^. A Bayesian neural network uses Bayesian inference to train a stochastic (or random) neural network^15,16^. Extensions of this approach include methods that employ variational inference^18^, dropout^19,20^, expectation propagation^21^, and stochastic gradient Markov chain Monte Carlo techniques^22^. Gaussian process-based models^17,23,24^ enable regression to a flexible function (e.g., in a regression problem) and can output a confidence interval of the predicted value in the output. These models have, for instance, been used to handle data with multiple levels of fidelity ^25^. Prediction models based on deep ensembles^26^, bootstrapping^27^, and deterministic uncertainty estimation using radial basis functions^28^ have also been proposed.

An alternative approach for integrating uncertainty into deep learning is evidential deep learning (EDL), which was proposed by Sensoy et al.^29^ and explicitly expresses the uncertainty of the prediction category by combining subjective logic with a neural network. Evidential deep learning has been employed in many fields, including medical image analysis^30,31^, target recognition in autonomous driving^32^, action recognition^33^, stereo matching^34^, and molecular discovery^35^.

However, to calculate the uncertainty, EDL uses the mean value of the (*K* – 1)-dimensional Dirichlet (or multivariate probability) distribution with parameter *α*_*k*_, where *K* is the number of classes included in the training data. This leads to the following two problems: first, EDL calculates the belief mass (uncertainty mass) *b*_u_ for the uncertain class; that is, data whose class is unknown by the network (henceforth, known as class u), and the probability *p*_u_ of class u is not output. In other words, the output is “the predicted class is *k* with uncertainty *b*_u_,” and it is ultimately at the discretion of the model user to determine what value of *b*_u_ means the result is trustworthy. Second, EDL assumes that the input always belongs to one of the* K* classes. That is, the output consists of predictions for each class *k* predicting whether the input belongs to that class, and the uncertainty of each prediction. This is true even for unexpected input data that does not belong to any known class. Examples of such data are outliers that cannot be correctly labeled at the time of training data labeling but are registered as “unknown” for the time being (called “contaminated data” here).

To address these problems, I propose a modified EDL (m-EDL) model that provides an output that predicts whether the input belongs to class u and not class *k* along with the probability for all *K* + 1 classes. Consequently, there is no need to determine a threshold at which the user judges the result to be uncertain. Moreover, when the output predicts that the instance belongs to a certain class *k*, the uncertainty of the prediction is nevertheless available. Finally, in contrast to the training data for EDL, the training data for m-EDL can include instances from class u. Several out-of-distribution (OOD) and open-set learning methods add a class to handle uncertainty^11,36^. In open-set recognition, Neal et al.^37^ augmented a dataset with a class of “counterfactual” images. Others explicitly train the classifier with a class of OOD samples near the in-distribution boundary^38^. By contrast, this study does not create an entirely new unknown class and attempt to learn it. Instead, the proposed m-EDL outputs in a natural way the “uncertainty of prediction” that EDLs naturally generate, and uses it as the probability of an unknown class. Only with this simple extension can data, including unknown classes, which EDL cannot handle, be learned. Moreover, the arbitrariness of the threshold, which is a weak point of EDL, can be resolved. In fact, the results of this study show the potential for improving the performance in discriminating unknown classes in test data without having to learn the counterfactual or OOD samples that existing approaches require.

The remainder of this paper is organized as follows. "Overview of the proposed model" explains the structure of the proposed m-EDL prediction model and compares it with that of EDL^29^. Additionally, a method for calculating the parameters in m-EDL is introduced and the likelihood calculation method used to train the model is explained. In "Advantages of m-EDL", the advantages of m-EDL modifications are explained. "Results" presents the experimental results, "Discussion" discusses the results, and "Methods" presents the methods used in the experiments.

## Overview of the proposed model

In this section, I first review the structure of EDL and then present m-EDL.

### EDL

**Figure 1:**
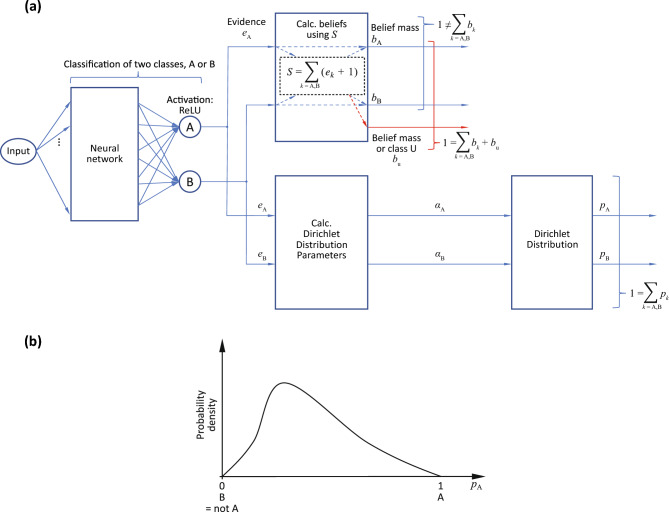
Conventional EDL^29^. (**a**) Overall structure (K = 2 classes). (**b**) Dirichlet distribution output.

I describe EDL^29^ using the two-class example shown in Fig. 1a. In this figure, the number of classes *K* is two (classes A and B); that is $k \in \{\mathrm{A,B}\}$.

First, the input is fed to a neural network, and evidence *e*_A_ and *e*_B_ for classes A and B respectively are obtained from its output, which is greater than or equal to zero. To train the neural network, Sensoy et al. employed the likelihood function using the sum-of-squares loss to stabilize neural network training^29^. The likelihood is calculated as follows:

1 $${\mathcal{L}}_{i}(\Theta )=\int \| {{\varvec{y}}}_{i}-{{\varvec{p}}}_{i}\|_{2}^{2}\frac{1}{B(\boldsymbol{\alpha })}\prod_{j}{p}_{ij}^{({\alpha }_{ij}-1)}d{{\varvec{p}}}_{i}=\sum_{j}{\bf E}[{y}_{ij}^{2}-2{y}_{ij}{p}_{ij}+{p}_{ij}^{2}].$$

Here, ${\varvec{p}}=({p}_{1},{ p}_{2}, \dots , {p}_{K})$ represents the probabilities for class *k*, *y* is 0 or 1 for each class, and *B*(*α*) is the beta function for the parameter ${\alpha }_{k}$, $k\in \{1, \dots , K\}$. Sensoy et al. also employed a Kullback–Leibler divergence (or relative entropy and I-divergence) term to regularize the predictive distribution by penalizing the divergences from class u^29^.

The belief mass *b*_*k*_ is obtained from the output of the neural network (evidence ${e}_{k}$ for each class *k*). In this example, the belief masses *b*_A_ and *b*_B_ are obtained using *S*, where $S={\sum }_{k=\mathrm{A,B}}({e}_{k}+1)$. The belief mass for each class *k* is calculated as follows:

2 $${b}_{k}=\frac{{e}_{k}}{S}=\frac{{e}_{k}}{\sum_{k}\left({e}_{k}+1\right)}.$$

Furthermore, the belief mass ${b}_{\rm{u}}$ for class u is calculated such that $1={\sum }_{k=\mathrm{A,B}}{b}_{k}+{b}_{\rm{u}}$ is satisfied.

The output from the EDL model is a Dirichlet distribution in *K* − 1 dimensions. A general Dirichlet distribution ${\varvec{p}}= \left({p}_{1},{p}_{2},\ldots ,{p}_{K}\right)$ with *K* parameters (${\alpha }_{k}$, $k\in \{1,\cdots ,K\}$) is given by the following equation:

3 $$D({\varvec{p}}|\varvec{\alpha })=\frac{1}{B(\varvec{\alpha })}\left\{\prod_{k=1}^{K}{p}_{k}^{{\alpha }_{k}-1}\right\}.$$

Similar to the belief masses *b*_*k*_, the Dirichlet distribution parameters ${\alpha }_{k}$ are obtained from the evidence *e*_*k*_ for each class *k* from the neural network using ${{\alpha }_{k}=e}_{k}+1$. These ${\alpha }_{k}$ parameters are directly used for the Dirichlet distribution. By contrast, the *b*_*k*_ are used to check the uncertainty (*b*_u_) and are not used for the distribution. However, Eq. (2) reveals that the Dirichlet distribution parameters and belief masses are related, as follows:

4 $${b}_{k}=\frac{{e}_{k}}{S}=\frac{{e}_{k}}{\sum_{k}\left({e}_{k}+1\right)}=\frac{{\alpha }_{k}-1}{\sum_{k}{\alpha }_{k}}.$$

For the example in Fig. 1a , *K* − 1 = 1 dimension, as shown in Fig. 1b. The probability distributions *p*_A_ and *p*_B_ for each class (A, B) are obtained using the Dirichlet distribution parameters *α*_A_ and *α*_B_, and the condition $1={\sum }_{k=\mathrm{A},\mathrm{B}}{p}_{k}$ is satisfied. For this example, the result obtained from the Dirichlet distribution is that the expected value that the input belongs to class A is $\overline{{p }_{\rm{A}}}= 20\%$, the expected value that the input belongs to class B is $\overline{{p }_{\rm{B}}}= 80\%$, and the uncertainty of this overall result (*b*_u_) is 30%. The sum of the expected values (i.e., 20% + 80%) satisfies the condition $1={\sum }_{k=\mathrm{A},\mathrm{B}}\overline{{p }_{k}}$. Note that the value of *b*_u_ is not included in this sum.

### m-EDL

In the proposed m-EDL, an additional class u is added to the original EDL to represent instances that do not belong to a known class. In this section, the extensions needed to EDL to obtain m-EDL are presented.

To obtain evidence from the neural network for all classes, including class u, the likelihood calculation must be extended. Equation (1) is extended from $j\in \{1,\cdots ,K\}$ to ${j}^{+}\in \{1,\cdots ,K,{\text{u}}\}$ as follows. First, ${{\varvec{y}}}_{i}^{+}=\left({y}_{i1},{y}_{i2},\cdots ,{y}_{iK},{y}_{i{\text{u}}}\right)$ and ${p}_{i}^{+}=\left({p}_{i1},{p}_{i2},\ldots ,{p}_{iK},{p}_{i\mathrm{u}}\right)$ are used to extend Eq. (1) to the following:

5 $$\begin{aligned} {\mathcal{L}}_{i}^{+}(\Theta )&=\int \| {{\varvec{y}}}_{i}^{+}-{{\varvec{p}}}_{i}^{+}{\| }_{2}^{2}\frac{1}{B\left({\varvec{\alpha }}^{+}\right)}\prod_{{j}^{+}}{p}_{i{j}^{+}}^{\left({\alpha }_{i{j}^{+}}-1\right)}d{{\varvec{p}}}_{i}\\ &=\sum_{{j}^{+}}\mathbf{E}\left[{y}_{i{j}^{+}}^{2}-2{y}_{i{j}^{+}}{p}_{i{j}^{+}}+{p}_{i{j}^{+}}^{2}\right]\\ &=\sum_{{j}^{+}}\left\{{y}_{i{j}^{+}}^{2}-2{y}_{i{j}^{+}}\cdot \mathbf{E}[{p}_{i{j}^{+}}]+\mathbf{E}[{p}_{i{j}^{+}}^{2}]\right\}. \end{aligned}$$

Furthermore, using the relationship of $\mathbf{E}[{p}_{i{j}^{+}}^{2}]=\mathbf{E}[{p}_{i{j}^{+}}{]}^{2}+\mathrm{Var}({p}_{i{j}^{+}})$, Eq. (5) is transformed as follows:

6 $${\mathcal{L}}_{i}^{+}(\Theta )=\sum_{{j}^{+}}\left\{({y}_{i{j}^{+}}-\mathbf{E}[{p}_{i{j}^{+}}]{)}^{2}+\mathrm{Var}({p}_{i{j}^{+}})\right\}.$$

$\mathbf{E}[{p}_{i{j}^{+}}]$ is the expected value of the Dirichlet distribution $D({{\varvec{p}}}^{+}|{\varvec{\alpha }}^{+})$, and ${\text{Var}}\left({p}_{i{j}^{+}}\right)$ is its variance. The detailed calculations are provided in the Supplementary Information available online.

The proposed m-EDL uses a Dirichlet distribution in *K*-dimensions. To output the Dirichlet distribution as ${{\varvec{p}}}^{+}= \left({p}_{1},{p}_{2},\cdots ,{p}_{K},{p}_{\rm{u}}\right)$, the following extension is required after introducing ${\mathrm{\alpha }}_{\text{u}}$:

7 $$D({{\varvec{p}}}^{+}|{\varvec{\alpha }}^{+})=\frac{1}{B({\varvec{\alpha }}^{+})}\left\{\prod_{k=1}^{K}{p}_{k}^{{\alpha }_{k}-1}\right\}\times {p}_{\rm{u}}^{{\alpha }_{\rm{u}}-1}=\frac{1}{B({\varvec{\alpha }}^{+})}\prod_{{k}^{+}}{p}_{{k}^{+}}^{{\alpha }_{{k}^{+}}-1}.$$

Here, $${{\varvec{\upalpha}}}^{+}=\left({\alpha }_{1},{\alpha }_{2},\cdots ,{\alpha }_{K},{\alpha }_{\text{u}}\right)$$ and $${k}^{+}\in \{1,\cdots ,K,{\text{u}}\}$$.

To calculate ${\mathrm{\alpha }}_{\text{u}}$, I first use $S=\sum_{k=1}^{K}({e}_{k}+1)=\sum_{k=1}^{K}{\alpha }_{k}$ and focus on the relationship of ${b}_{\rm{u}}+\sum_{k=1}^{K}{b}_{k}=1$. These relationships should be satisfied using subjective logic^39^, where the Dempster–Shafer theory is used in the framework of the Dirichlet distribution.

From this point, the extension to class u begins. When ${b}_{\rm{u}}+\sum_{k=1}^{K}{b}_{k}=1$ is transformed using Eq. (2), it is expressed as follows:

8 $${b}_{\text{u}}=\frac{K}{\sum_{k}{\alpha }_{k}}.$$

If Eq. (2) is further extended to class u and written as ${b}_{\rm{u}}=\frac{{\alpha }_{\rm{u}}-1}{\sum_{k}{\alpha }_{k}}$, then

9 $${\mathrm{\alpha }}_{\text{u}}=K+1,$$

is obtained. Additionally, if ${b}_{k}$ is extended to class u based on ${b}_{k}=\frac{{e}_{k}}{S}$ in Eq. (2), then the belief mass of class u can be written as ${b}_{\rm{u}}=\frac{{e}_{\rm{u}}}{S}$. Hence, the evidence for class u can be written as follows:

10 $${e}_{\text{u}}=K.$$

Equations (8)–(10) are obtained by the extension to class u, but they are derived from the relationships between $S=\sum_{k=1}^{K}({e}_{k}+1)=\sum_{k=1}^{K}{\alpha }_{k}$ and ${b}_{\rm{u}}+\sum_{k=1}^{K}{b}_{k}=1$. Therefore, they are in line with the belief mass of the Dempster–Shafer theory and subjective logic^39^.

**Figure 2:**
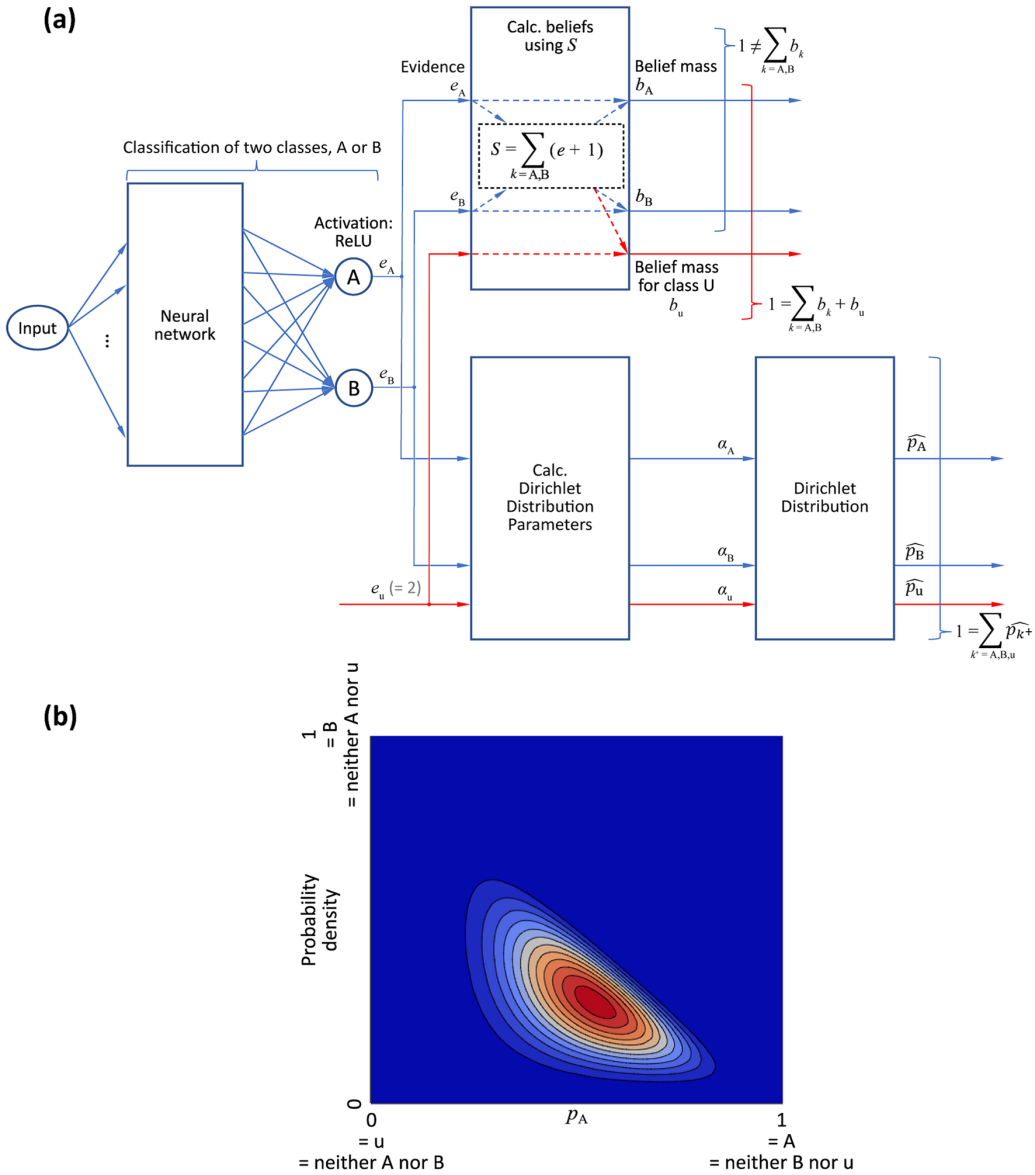
Proposed m-EDL. (**a**) Overall structure (*K* = 2 classes). (**b**) Dirichlet distribution output. The probability density increases from blue to red.

For the same two-class example used in "EDL", the structure of the proposed m-EDL is shown in Fig. 2a. In this example, $k \in \{\mathrm{A},\mathrm{ B}\}$; hence, ${k}^{+} \in \{\mathrm{A},\mathrm{ B},\mathrm{ u}\}$ is defined. As in EDL, the input is fed to the neural network, and evidence *e*_A_ and *e*_B_ for classes A and B are obtained from the output of the neural network. Next, belief masses *b*_A_ and *b*_B_ are obtained using *S* such that $S={\sum }_{k=\mathrm{A},\mathrm{B}}({e}_{k}+1)$. The belief mass ${b}_{\rm{u}}$ for class u is calculated using $1={\sum }_{k=\mathrm{A},\mathrm{B}}{b}_{k}+{b}_{\rm{u}}$. This ${b}_{\rm{u}}$ is used to obtain evidence *e*_u_ for class u, as described in detail above. The probability distributions *p*_A_, *p*_B_, and *p*_u_ for each class (A, B, and u) are obtained using the Dirichlet distribution parameters *α*_A_, *α*_B_, and *α*_u_. These distribution parameters are themselves obtained from the belief masses *b*_A_ and *b*_B_ as well as ${b}_{\rm{u}}$, and the condition $1={\sum }_{{k}^{+}=\mathrm{A},\mathrm{B},\mathrm{ u}}{p}_{{k}^{+}}$ is satisfied.

The output from m-EDL is a Dirichlet distribution in *K* dimensions (two dimensions), as shown in Fig. 2b, where the increase in probability density is indicated by hue from blue to red. Furthermore, the results from the Dirichlet distribution are the expected value that the input belongs to class A is $\widehat{{p}_{\rm{A}}}= 50\%$, the expected value that the input belongs to class B is $\widehat{{p}_{\rm{B}}}= 30\%$, and the expected value that the input belongs to class u (that is, the input cannot be said to belong to either class A or B) is $\widehat{{p}_{\rm{u}}}= 20\%$. The sum of these expected probabilities also satisfies $1={\sum }_{{k}^{+}=\mathrm{A},\mathrm{B},\mathrm{ u}}\widehat{{p}_{{k}^{+}}}$.

As explained in the Supplementary Information and illustrated in Supplementary Fig. S1 (both available online), the expected value $\overline{{p }_{k}}$ satisfying $1={\sum }_{k=\mathrm{A},\mathrm{B}}\overline{{p }_{k}}$ obtained in EDL can also be obtained from $\widehat{{p}_{{k}^{+}}}$. In the above example, $\overline{{p }_{\rm{A}}}= 62.5\%$ and $\overline{{p }_{\rm{B}}}= 37.5\%$.

## Advantages of m-EDL

There are two main advantages of m-EDL. First, it is unnecessary to determine the threshold at which the model user will judge the result to be uncertain. As described in "EDL", the EDL model of Sensoy et al.^29^ outputs the expected value that the input data class is class A, the expected value that the input data class is class B, and uncertainty ($\overline{{p }_{\rm{A}}}= 20\%$, $\overline{{p }_{\rm{B}}}= 80\%$, and uncertainty = 30%, respectively), whereas m-EDL outputs the expected value that the input data class is class A, the expected value that the input data class is class B, and the expected value that the input data class is class u; that is, that the input cannot be said to be either class A or B ($\widehat{{p}_{\rm{A}}}= 50\%$, $\widehat{{p}_{\rm{B}}}= 30\%$, and $\widehat{{p}_{\rm{u}}}= 20\%$, respectively).

The EDL model's output is in the form of input-data prediction classes and the corresponding uncertainty for each class. Hence, an uncertainty threshold must be set^29^ to determine whether the results should be used. The accuracy of the model changes according to this threshold^29^. In contrast, m-EDL has an output that includes the expected value for all *K* classes and class u. These probabilities sum to 1. Therefore, the user can simply choose the class with the highest probability from the* K* + 1 classes as the predicted class. It is unnecessary to define an uncertainty threshold in the first place. In addition, even when m-EDL predicts a certain *k* class from *K* classes, the uncertainty *b*_u_ is nevertheless available from m-EDL.

Furthermore, training data can include data from class u. I explain why this is the case below.

Here, the likelihood function used for the simple likelihood estimation in Sensoy et al.’s EDL^29^ for parameter fitting of the neural network part of EDL (as shown in Fig. 1a) is expressed as follows:

11 $${\mathcal{L}}_{i}(\Theta )=\sum_{j=1}^{K}[{y}_{ij}\{\mathrm{log}({S}_{i})-\mathrm{log}({\alpha }_{ij})\}],$$

where *y*_*i*_ is the one-hot vector encoding the ground-truth class of observation *x*_*i*_ with ${y}_{ij} = 1$ and ${y}_{ik} = 0$ for all $k\ne j,$ and where the *j*th class is the correct label for observation *i*. Meanwhile, ${\alpha }_{ij}$ indicates the *K* parameters of the Dirichlet distribution for observation *i* and ${S}_{i}={\sum }_{j=1}^{K}{\alpha }_{ik}$.

In Sensoy et al.’s method^29^, it is assumed that the input data belongs to one of the *K* classes; therefore, the range that index *j* can take is 1 through *K*.

By contrast, the m-EDL shown in Fig. 2a introduces the parameter ${\alpha }_{\rm{u}}$ of the Dirichlet distribution. That is, it is in the form of ${j}^{+} \in \{1,\cdots ,K,\mathrm{ u}\}$, which is an extension of $j \in \{1,\cdots ,K\}$. Applying this extension to the likelihood function of Eq. (11) results in the following, with ${j}^{+} \in \{1,\cdots ,K,\mathrm{ u}\}$:

12 $$\begin{aligned} {\mathcal{L}}_{i}^{+}(\Theta )&=\sum_{{j}^{+}}[{y}_{i{j}^{+}}\{\mathrm{log}({S}_{i})-\mathrm{log}({\alpha }_{i{j}^{+}})\}]\\ &=\sum_{j}[{y}_{ij}\{\mathrm{log}({S}_{i})-\mathrm{log}({\alpha }_{ij})\}]+{y}_{i\mathrm{u}}\{\mathrm{log}({S}_{i})-\mathrm{log}({\alpha }_{i\mathrm{u}})\}, \end{aligned}$$

where ${y}_{i{j}^{+}}$ is a one-hot vector that contains class u, indicating that the data labeled as belonging to class u can be included in the training data of m-EDL.

The implications of this extension are as follows. First, it becomes possible to learn a dataset that, for example, consists of handwritten digits 0–9 such as MNIST (ground truth labels 0–9) mixed with a completely different dataset type (correct label u or 10). Additionally, this learning may help determine the accuracy of predictions about whether, for example, the input is a digit from 0 to 9 or is not a digit when non-numeric data are mixed into the test dataset.

## Results

I investigated whether m-EDL has the same performance as EDL through comparative experiments. I also investigated whether m-EDL has an advantage when including class u in the training data. The objective of this evaluation was to determine the following:

(Q1): whether the use of m-EDL reduces the prediction accuracy for a class *k* when the same training and test data are given to EDL and m-EDL models;

(Q2): whether a) an m-EDL model that has learned class u has the same prediction accuracy for a class *k* when compared with an EDL model that cannot learn class u, and b) m-EDL predicts class u with higher accuracy than EDL;

(Q3): if the ratio of class u data included in the training data affects the accuracy of predicting classes *k* and u in the test data;

(Q4): what happens when the properties of class u data that are blended with the training data and test data in Q2 and Q3 are exactly the same.

To answer these questions, several datasets and models were prepared. Conditions that depended on whether data from class u were included in the training and/or test data, as well as which model was used to learn the data, were used in the evaluation.

### Performance comparison of EDL and m-EDL on class k data (Q1)

Here, I evaluate whether the performance of m-EDL is comparable to that of EDL in the situation assumed by EDL; that is, the situation where all training and test data belong to class *k*. In other words, both the training and test data were composed only of images from MNIST, and the following two conditions were compared: (1) the EDL model trained and tested on datasets with no class u data and (2) the m-EDL model trained and tested on datasets with no class u data.

**Figure 3:**
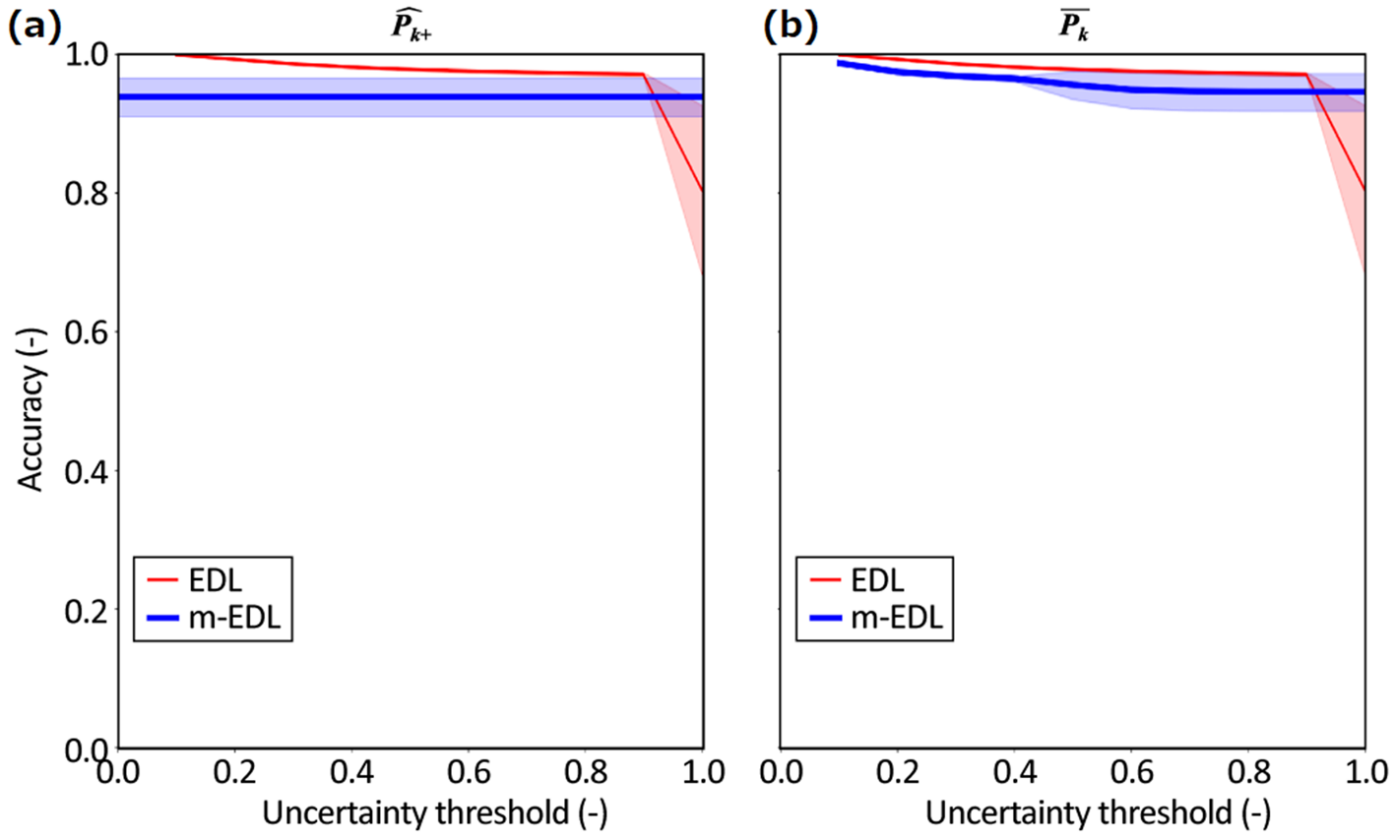
Accuracy of EDL and m-EDL when both the training and test datasets contain no class u data. (**a**) Results when $\widehat{{{\varvec{p}}}_{{{\varvec{k}}}^{+}}}$ is used in m-EDL classification. (**b**) Results when $\overline{{{\varvec{p}} }_{{\varvec{k}}}}$ is converted from $\widehat{{{\varvec{p}}}_{{{\varvec{k}}}^{+}}}$ and used in m-EDL classification with the same uncertainty threshold as that of EDL.

Figure 3 compares the accuracies of EDL (thin solid red line) and m-EDL (thick solid blue line). Each line shows the mean value and the shaded areas indicate the standard deviation. The accuracy of EDL changes with respect to each uncertainty threshold; the accuracy is plotted on the vertical axis with the uncertainty threshold indicated by the horizontal axis. The accuracy of EDL improves as the threshold decreases because only a classification result the model is confident of is treated as a classification result. Figure 3a shows the results when $\widehat{{{\varvec{p}}}_{{{\varvec{k}}}^{+}}}$ is used for the classification results of m-EDL. An uncertainty threshold is not used for the classification result of m-EDL; a result parallel to the horizontal axis is obtained. In contrast, Fig. 3b shows the results when $\widehat{{{\varvec{p}}}_{{{\varvec{k}}}^{+}}}$ is converted to $\overline{{{\varvec{p}} }_{{\varvec{k}}}}$ and the uncertainty threshold used for EDL is also used for m-EDL.

These graphs show that the accuracy of m-EDL is lower than that of EDL, except in the region where the uncertainty threshold is 0.9 or more. However, no substantial decrease in accuracy is observed, and it can be said that the performance of m-EDL would be sufficient depending on the application.

### Performance comparison of EDL and m-EDL when class u is included in the training and test data (Q2)

In this experiment, the properties of the class u data that are included in the training and test data are completely different; that is, they are obtained from different datasets. This makes it possible to confirm whether the learned uncertain class features are regarded as features that are not class *k* rather than features that are class u learned during training.

First, I consider whether an m-EDL model that has learned class u has the same prediction accuracy for class *k* when compared with an EDL model that cannot learn class u (Q2a). I then consider whether it can determine class u with higher prediction accuracy (Q2b).

**Figure 4:**
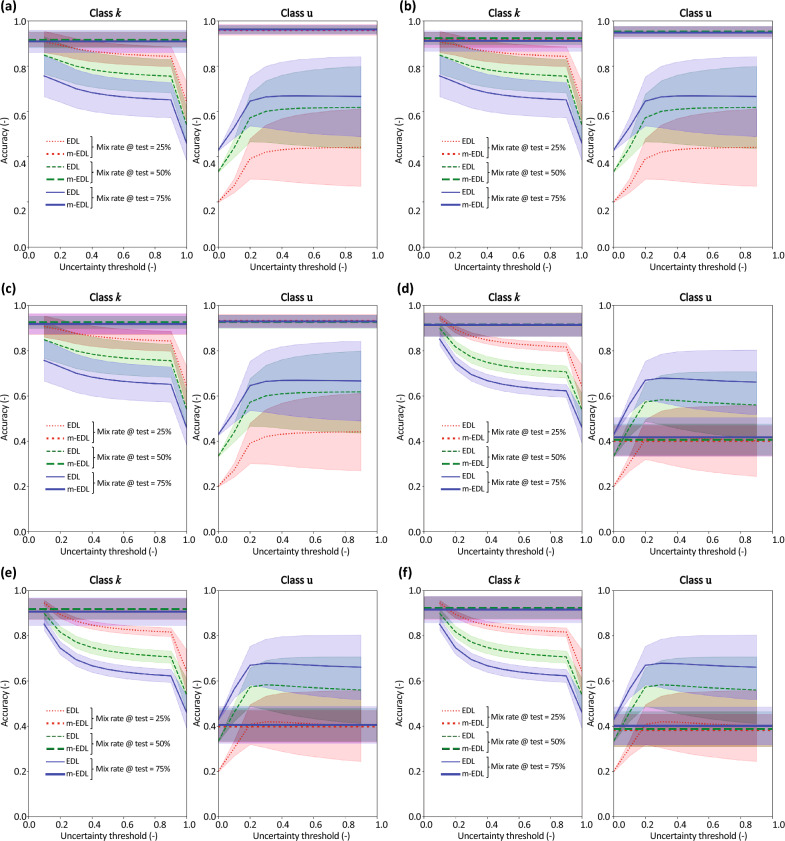
Accuracy comparison of EDL and m-EDL. Line colors indicate the proportion of class u in the test data, and top and bottom plots show the accuracy for class *k* data and class u data, respectively. Results when m-EDL has learned class u (EMNIST data) but is tested on Fashion MNIST data for class *u* mix rates in the training data of (**a**) 25%, (**b**) 50%, and (**c**) 75%. These are percentages of the number of MNIST data. Results when m-EDL has learned class u (Fashion MNIST data) but is tested on EMNIST data for class *u* mix rates in the training data of (**d**) 25%, (**e**) 50%, and (**f**) 75%.

The following two cases are considered: (1) EDL is tested on data that include Fashion MNIST data, and m-EDL is trained on data that include EMNIST data, but tested on data that include Fashion MNIST data. Figure 4a–c shows the results for class u rates of 25%, 50%, and 75% in training data, respectively. The lines of different colors indicate the results for class u rates of 25%, 50%, and 75% in the test data (1–2). These are percentages of the number of MNIST data. Additionally, Table 1 presents the mean accuracies of EDL and mEDL for each condition. (2) EDL is tested on data that include EMNIST data, and m-EDL is trained on data that include Fashion MNIST data, but tested on data that include EMNIST data. Figure 4d–f shows the results for class u rates of 25%, 50%, and 75% in the training data, respectively. The lines of different colors indicate the results for class u rates of 25%, 50%, and 75% in test data. These are percentages of the number of MNIST data. Additionally, Table 2 presents the mean accuracies of EDL and mEDL for each condition.

**Table 1:** Accuracy comparison of EDL and m-EDL. These values are mean accuracy through the uncertainty threshold. This table corresponds to Fig. 4a–c.

Training: MNIST + EMNIST Test: MNIST + FashionMNIST (Fig. 4a–c)			Avg. accuracy for *k*		Avg. accuracy for u	
			EDL	mEDL	EDL	mEDL
Mix rate in training data: 25%	Mix rate in test data	25%	0.842	0.917	0.39	0.931
		50%	0.791	0.925	0.563	0.927
		75%	0.662	0.917	0.627	0.928
Mix rate in training data: 50%	Mix rate in test data	25%	0.842	0.916	0.390	0.951
		50%	0.761	0.922	0.563	0.952
		75%	0.662	0.910	0.627	0.948
Mix rate in training data: 75%	Mix rate in test data	25%	0.842	0.916	0.390	0.958
		50%	0.761	0.915	0.563	0.961
		75%	0.662	0.909	0.627	0.960

**Table 2:** Accuracy comparison of EDL and m-EDL.

Training: MNIST + FashionMNIST Test: MNIST + EMNIST (Fig. 4d–f)			Avg. accuracy for *k*		Avg. accuracy for u	
			EDL	mEDL	EDL	mEDL
Mix rate in training data: 25%	Mix rate in test data	25%	0.831	0.921	0.378	0.381
		50%	0.736	0.922	0.536	0.386
		75%	0.659	0.914	0.634	0.400
Mix rate in training data: 50%	Mix rate in test data	25%	0.831	0.917	0.378	0.397
		50%	0.736	0.918	0.536	0.405
		75%	0.659	0.906	0.634	0.405
Mix rate in training data: 75%	Mix rate in test data	25%	0.831	0.916	0.378	0.401
		50%	0.736	0.915	0.536	0.406
		75%	0.659	0.912	0.634	0.417

These values are mean accuracy through the uncertainty threshold. This table corresponds to Fig. 4d–f.

Under these two conditions, the one-hot vector *y*_*j*_ of the data has *K* = 10 dimensions. Therefore, all elements of the one-hot vectors of class u (EMNIST or Fashion MNIST data) in the test data were set to 0. In each of the following cases, the same processing was applied when EDL was tested on data including class u data.

The left plots of Fig. 4a–c and Table 1 (avg. accuracy for *k*) show the results for class *k* data for the first condition. The line color indicates the ratio of the class u data included in the test data, and it is assumed that the accuracy decreases as the mix ratio of class u in the test data increases. The results show that the accuracy of m-EDL with respect to class *k* is high and robust for the mix rate of class u in the training and test data: it can be seen from the left plots in Fig. 4a–c that when the m-EDL model that has learned class u is compared with the EDL model, which cannot learn class u, it has equal or higher accuracy with respect to class *k*. Moreover, the accuracy of m-EDL is not easily affected by the ratio of class u in the test data as well as the training data.

The right plots of Fig. 4a–c and Table 1 (avg. accuracy for u) show the accuracy for class u data, that is, the accuracy that the “data that was judged as ‘I do not know’ is actually different from the data classes learned so far.” The right plots of Fig. 4a–c show that the accuracy of m-EDL with respect to class u is high and robust for the mix rate of class u in the training and test data. It is natural to increase the accuracy for class u of EDL when the ratio of class u increases because the accuracy increases when the ratio of class u increases even if class u is randomly classified via EDL.

Figure 4d–f and Table 2 (avg. accuracy for *k*) show the results for the second condition, which is exactly the same as the first condition except that the EMNIST and Fashion MNIST datasets switch roles. Again, the accuracy of m-EDL with respect to class *k* is high and robust, as in the left plots of Fig. 4a–c. The results in the left plots of Fig. 4d–f reveal that the m-EDL model that learned class u, when compared with EDL, achieved an equal or higher accuracy with respect to class *k*, and the accuracy of m-EDL was not easily affected by the ratio of class u in the test and training data.

However, the right plots of Fig. 4d–f and Table 2 (avg. accuracy for u) show that the accuracy of m-EDL with respect to class u cannot be said to be better than that of EDL.

### Effect of the ratio of the class u included in the training data on the prediction accuracy of classes k and u in the test dataset (Q3)

**Figure 5:**
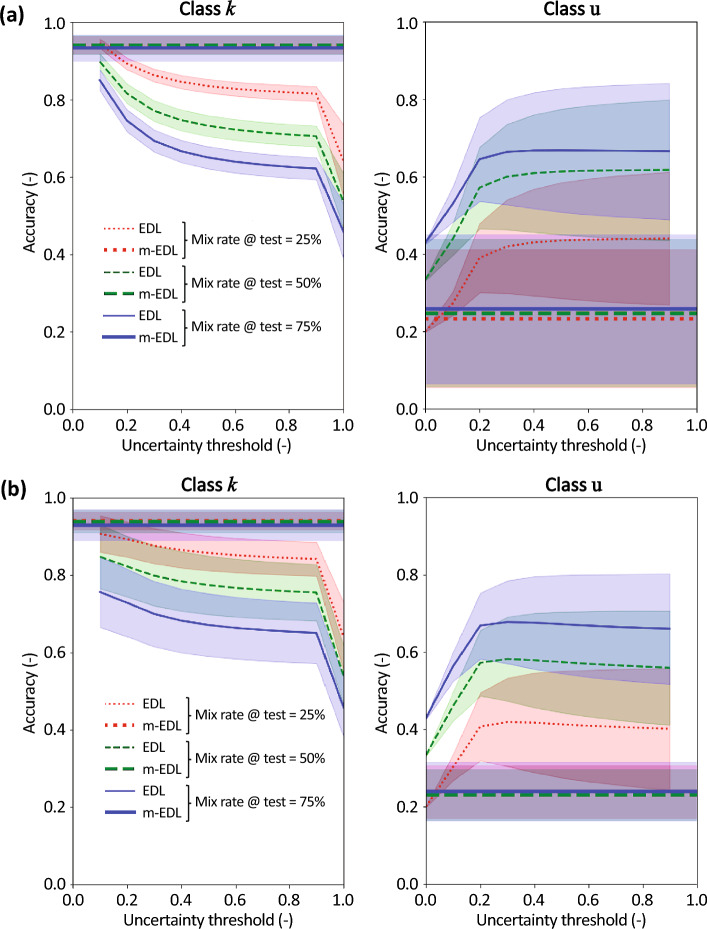
Accuracy comparison of EDL and m-EDL when neither EDL nor m-EDL have learned class *u*. Line colors indicate the mix rate of class u in the test data, and left and right plots show the accuracy for class k data and class u data, respectively. (**a**) Results for Fashion MNIST data. (**b**) Results for EMNIST data.

In the comparison of the two patterns in "Performance comparison of EDL and m-EDL when class u is included in the training and test data (Q2)", if the ratio of class u in the training data affects the prediction accuracy of the class *k* and u data, then the ratio of class u included in the training data must be appropriately selected. To answer whether this is the case, I used the results from "Performance comparison of EDL and m-EDL when class u is included in the training and test data (Q2)" (Fig. 4a–c and d–f, which have training data mix ratios of 25%, 50%, and 75%, respectively), and added the following two cases:1) Fashion MNIST is included in the test data, but neither EDL nor m-EDL are trained on class u data (a training data mix ratio of 0%; Fig. 5a) and 2) EMNIST is included in the test data, but neither EDL nor m-EDL are trained on class u data (a training data mix ratio of 0%; Fig. 5b). The lines of different colors indicate the results for class u rates of 25%, 50%, and 75% in the test data.

In the left plot of Fig. 5a, the accuracy improved for class *k* as shown in the left plots of Fig. 4a–c, whereas in the right plot of Fig. 5a, there was no improvement in accuracy for class u. In the right plots of Fig. 4a–c, the accuracy for class u was improved even when the ratio of class u in the training data was small. These results suggest that the accuracy for class u may be improved by having m-EDL learn even a small amount of class u data. Moreover, there is no particular need for these data to be related to the class u data in the test data.

The right plot of Fig. 5b shows that m-EDL did not lead to improvements in accuracy for class u. Moreover, in the right plots of Fig. 4d–f, the accuracy of m-EDL for class u is not better than that of EDL; however, when compared with the results in the right plot of Fig. 5b, it is clear that the accuracy of m-EDL for class u is improved even if the ratio of class u in the training data is small.

It can be inferred from these comparisons that the amount of accuracy improvement for class u changes depending on the characteristics of class u in the training and test data.

### Impact of the nature of class u in the training and test data (Q4)

As shown in "Performance comparison of EDL and m-EDL when class u is included in the training and test data (Q2)" and "Effect of the ratio of the class u included in the training data on the prediction accuracy of classes k and u in the test dataset (Q3)", the amount of improvement in accuracy for class u data changes depending on the characteristics of u in the training data and test data. Hence, I evaluated whether the accuracy for class u always improves when the characteristics of u in the training and test data are exactly the same (i.e., when the class u data are from the same dataset).

**Figure 6:**
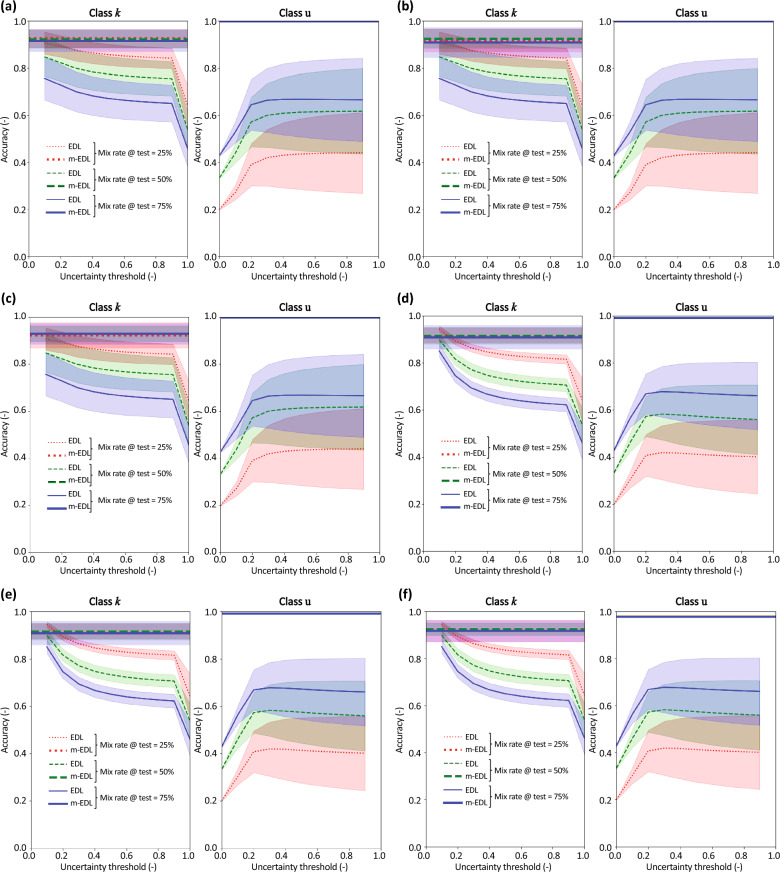
Accuracy comparison of EDL and m-EDL. Line colors indicate the proportion of class u in the test data, and top and bottom plots show the accuracy for class *k* data and class u data, respectively. Results when m-EDL has learned class u (Fashion MNIST) for class *u* mix rates in the training data of (**a**) 25%, (**b**) 50%, and (**c**) 75%. These are percentages of the number of MNIST data. Results when m-EDL has learned class u (EMNIST)for class *u* mix rates in the training data of (**d**) 25%, (**e**) 50%, and (**f**) 75%.

The following two conditions were considered: (1) when Fashion MNIST is included in both the test and training data [Fig. 6a–c and Table 3 (avg. accuracy for *k* and u)] and (2) when EMNIST is included in both the test and training data [Fig. 6d–f and Table 4 (avg. accuracy for *k* and u)].

**Table 3:** Accuracy comparison of EDL and m-EDL.

Training: MNIST + FashionMNIST Test: MNIST + FashionMNIST (Fig. 6a–c)			Avg. accuracy for *k*		Avg. accuracy for u	
			EDL	mEDL	EDL	mEDL
Mix rate in training data: 25%	Mix rate in test data	25%	0.842	0.922	0.390	0.998
		50%	0.761	0.929	0.563	0.997
		75%	0.662	0.928	0.627	0.997
Mix rate in training data: 50%	Mix rate in test data	25%	0.842	0.917	0.390	0.998
		50%	0.791	0.925	0.563	0.998
		75%	0.662	0.909	0.627	0.998
Mix rate in training data: 75%	Mix rate in test data	25%	0.842	0.926	0.390	0.999
		50%	0.761	0.923	0.563	0.999
		75%	0.662	0.916	0.627	0.999

These values are mean accuracy through the uncertainty threshold. This table corresponds to Fig. 6a–c.

**Table 4:** Accuracy comparison of EDL and m-EDL.

Training: MNIST + EMNIST Test: MNIST + EMNIST (Fig. 6d–f)			Avg. accuracy for *k*		Avg. accuracy for u	
			EDL	mEDL	EDL	mEDL
Mix rate in training data: 25%	Mix rate in test data	25%	0.831	0.917	0.378	0.978
		50%	0.736	0.925	0.536	0.978
		75%	0.659	0.917	0.634	0.978
Mix rate in training data: 50%	Mix rate in test data	25%	0.831	0.916	0.378	0.989
		50%	0.736	0.922	0.536	0.988
		75%	0.659	0.910	0.634	0.988
Mix rate in training data: 75%	Mix rate in test data	25%	0.831	0.916	0.378	0.992
		50%	0.736	0.915	0.536	0.992
		75%	0.659	0.909	0.634	0.992

These values are mean accuracy through the uncertainty threshold. This table corresponds to Fig. 6d–f.

The differences in Fig. 6a–c and d–f are the mix rates of class u in the training data (25%, 50%, and 75%, respectively). The lines of different colors indicate the results for class u rates of 25%, 50%, and 75% in the test data. These are percentages of the number of MNIST data. In particular, the right-hand side plots of Fig. 6a–f confirm that the accuracy of m-EDL is higher than that in the cases considered for Q2 and Q3 and is almost 100%.

In the cases of Q2 and Q3, the class u data in the training and/or test data have different characteristics, and the accuracy of m-EDL on the class u data changed depending on the combination. Meanwhile, in the Q4 cases, class u data had the same characteristics during both training and testing, and hence, the accuracy is very high. From this, it is clear that the feature learning of class u in the training data contributes to the improvement in accuracy that m-EDL exhibits when learning class u. However, in the comparisons of Q2, particularly when m-EDL was trained using EMNIST and both EDL and m-EDL were tested on data including Fashion MNIST, examples can be found where the accuracy improved even when the unknown classes in the training and test data differ. Therefore, m-EDL has the potential to improve accuracy by excluding uncertain data as a result of learning unrelated data that do not belong to class *k* data, although this depends on the combination of class u data in the training and test data.

Here, we hypothesize regarding the combination of class u datasets to be mixed during training that will increase the class u accuracy in testing. The hypothesis is that “if class u data whose characteristics are as close as possible to those of class *k* are learned during training, class u data in the test can be discriminated as class u as long as the characteristics of class u given during the test are different from those in training”; i.e., “if a boundary that can distinguish the range of class *k* more strictly with u whose characteristics are close to those of class *k* is learned via mEDL, class u can be easily distinguished.” Conversely, “if the class u data during training are far from the characteristics of *k*, the decision boundary between *k* and u is freely determined, and if the class u data in the test are close to *k*, they may be incorrectly classified.”

To test this hypothesis, I introduced another dataset (Cifar-10^40^) and evaluated the similarity of the characteristics of different datasets. The Cifar-10 dataset used had images of 28 × 28 pixels for similarity calculation (consistent with the other dataset), which were grayscaled using a previously proposed method^41^. Table 5 presents the similarity of MNIST, EMNIST, Fashion-MNIST, and Cifar-10. Here, the structural similarity (SSIM) was determined by randomly selecting 500,000 images of the datasets to be compared, and the mean and variance were calculated as the similarity between the datasets.

**Table 5:** Mean (standard deviation) values of the structural similarity between datasets.

	MNIST	Fashion-MNIST	EMNIST	Cifar-10
MNIST	–	0.123 (0.094)	0.149 (0.116)	0.012 (0.048)
Fashion-MNIST	–	–	0.046 (0.067)	0.046 (0.067)
EMNIST	–	–	–	0.014 (0.056)
Cifar-10	–	–	–	–

**Figure 7:**
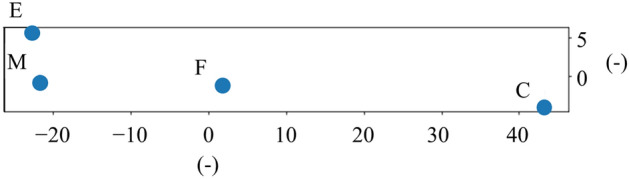
Location of each dataset estimated via MDS, where the points M, F, E, and C represent the locations of the MNIST, Fashion-MNIST, EMNIST, and Cifar-10 datasets, respectively, and the distance between points is proportional to the inverse of the similarity. The numbers on the horizontal and vertical axes are dimensionless.

The distance between datasets was determined as the inverse of the SSIM, and the positional relationship of the datasets on a two-dimensional plane was estimated via multidimensional scaling (MDS)^41^, as shown in Fig. 7.

As shown in Fig. 7, EMNIST was more similar to Fashion-MNIST than to EMNIST. The newly introduced Cifar-10 is an image dataset with characteristics that are more different from those of MNIST than those of both EMNIST and Fashion-MNIST. The hypothesis explains the result presented in "Performance comparison of EDL and m-EDL when class u is included in the training and test data (Q2)" that the accuracy of class u was higher in Case 1 when u was trained with EMNIST and classified with test data containing Fashion MNIST than in Case 2 when u was trained with Fashion-MNIST and classified with test data containing EMNIST. The reason why the accuracy of class u was higher in Case 1 is because the characteristics of EMNIST were closer than those of Fashion-MNIST to the those of MNIST. mEDL-trained EMNIST was able to identify Fashion-MNIST, which was given during testing and had more distant characteristics than EMNIST, as class u. To verify this hypothesis, I compared the accuracy of class u in Case 3, where class u was trained with Cifar-10 and was classified with the test data containing EMNIST, with those for Cases 1 and 2. If the hypothesis is correct, the accuracy of class u should decrease in the following order: Case 1 > Case 2 > Case 3.

Table 6 presents the accuracies of mEDL for class u in each case. Indeed, the accuracy of Case 3 was the lowest, suggesting that “if class u has characteristics close to those of class *k* during training, class u in the test can be detected as class u as long as the characteristics of class u given during testing are farther than those in the training.”

**Table 6:** Comparison of the accuracies of mEDL for class u in different cases.

			Accuracy of mEDL for class u		
			Case 1 (Fig. 4a–c): Training: MNIST + EMNIST Test: MNIST + Fashion	Case 2 (Fig. 4d–f): Training: MNIST + Fashion Test: MNIST + EMNIST	Case 3: Training: MNIST + Cifar-10 Test: MNIST + EMNIST
Mix rate in training data: 25%	Mix rate in test data	25%	0.931	0.381	0.283
		50%	0.927	0.386	0.289
		75%	0.928	0.400	0.297
Mix rate in training data: 50%	Mix rate in test data	25%	0.951	0.397	0.323
		50%	0.952	0.405	0.305
		75%	0.948	0.405	0.313
Mix rate in training data: 75%	Mix rate in test data	25%	0.958	0.401	0.309
		50%	0.961	0.406	0.301
		75%	0.960	0.417	0.298

## Discussion

Deep learning has led to many remarkable advances; however, in many scenarios, the uncertainty of the model output is required. EDL is one model that can provide this uncertainty. In this study, I proposed a method that extends the EDL model proposed by Sensoy et al.^29^ to predict that the input belongs to class u and not *k* along with a probability and evaluated its performance and behavior.

The proposed m-EDL does not require the user to set a threshold for the uncertainty to interpret the results. Because m-EDL does not require this parameter, the accuracy of the model is not affected by its value. Additionally, m-EDL allows data belonging to unknown classes to be included in the training dataset.

The results of the experiments revealed that m-EDL performs comparably to EDL when there are no instances of unknown classes. When there are instances of unknown classes, m-EDL performs better than EDL on known classes. Its performance in class u improves depending on the combination of unknown data in the training and test data. m-EDL can learn the characteristics of class u itself, and it has the potential to predict unknown classes even when the unknown classes in the training data and test data have different properties.

The accuracy of m-EDL on class u changed depending on the combination of classes in the data.

The additional analysis with the Cifar-10 dataset indicated that during training, if class u, whose characteristics evaluated via the SSIM are as close as possible to the characteristics of class *k*, is learned, the class u data in the test can be determined as class u as long as the characteristics of class u in testing are farther than those in training. From the above results, if class u is to be mixed intentionally during training to increase the discrimination accuracy of class u in mEDL, it is necessary that the characteristics of the mixed u data are as close as possible to those of class *k*.

In this study, I set the class *k* data to MNIST data. In future research, it is necessary to determine that the optimized mEDL exhibits superior performance for various datasets.

## Methods

The datasets MNIST^42^, Fashion MNIST^43^, and EMNIST^44^ were used in the evaluation. MNIST was used to provide the data for class *k*. It consists of images of handwritten digits. Each image is labeled as belonging to classes 0–9; that is,* K* = 10.

The data for class u were obtained from either Fashion MNIST or EMNIST according to the experiment. Fashion MNIST is a dataset of 60,000 28 × 28 grayscale images of ten fashion categories (“t-shirt/top,” “trousers,” “pullover,” “dress,” “coat,” “sandal,” “shirt,” “sneaker,” “bag,” or “ankle boot”) along with a test set of 10,000 images. All the images from this dataset were categorized as class u in this evaluation. Therefore, even if images of a t-shirt or dress appear in the training or test data, the correct label for both images is class u. The EMNIST dataset is a set of handwritten character digits derived from the NIST Special Database 19 and converted to a 28 × 28 pixel image format and dataset structure that directly matches the format of the MNIST dataset. Specifically, I used EMNIST Letters, i.e., 26 capital letters (26 classes). They were all categorized as class u. Therefore, even if images of “A,” “C,” or “X” exist in the training or test data, the correct label is u.

The total number of training data was 60,000. When blending class u (from EMNIST and/or Fashion MNIST) into the MNIST data, the class u data to be blended were randomly selected prior to blending. The total number of test data was 10,000. The class u blending method was the same as that used for the training data.

A fully coupled neural network was constructed in Python using the Keras library to build the neural networks used for the EDL and m-EDL models. The input image was a 28 × 28 grayscale normalized image, and there were two hidden layers with 32 dimensions each. The size of the output layer was *K* (= 10) or *K* + 1 (= 11). The activation function was ReLU, and Adam was used for the optimization. Mini-batch learning was used with a batch size of 64, initial learning rate of 10^−3^, and no decay.

Learning involved early stopping, with a maximum number of epochs of 100. Convergence was confirmed in all cases. The data used for validation consisted of 10% of the training data. The training and testing were repeated 100 times for each condition in the evaluation, and the mean and standard deviation of the accuracy are reported.

The experiments were run on a computer equipped with an Intel Core i7-7800X, NVIDIA GeForce RTX 2080 SUPER, 32 GB of RAM, and a Windows 10 operating system.

### Supplementary Information

Supplementary Information.

## Data Availability

Data for this study are available from the corresponding author on reasonable request.

## Code availability

The code used in this study is available on GitHub at https://github.com/naga0862/m-EDL.
