# Supplementary material for: Learning and predicting the unknown class using evidential deep learning
Source: Sci Rep. 2023 Sep 9;13:14904. doi: 10.1038/s41598-023-40649-w (PMC10492799; doi:10.1038/s41598-023-40649-w)
Supplement: Supplementary file 1 — Supplementary Information. [file 41598_2023_40649_MOESM1_ESM.pdf]

Supplementary Information 1

Learning and predicting the unknown class using evidential deep learning

Akihito Nagahama

The University of Electro-Communications, 1-5-1 Chofugaoka, Chofu, Tokyo 182-8585, Japan. ORCID: 0000-0001-7285-5110

Calculation of the expected value of the Dirichlet distribution and its variance

I calculate $E\left[p_{ij^+}\right]$ and $Var\left(p_{ij^+}\right)$, which are needed for the likelihood calculation in equation (6), as follows.

Here, the expected value $E[p_{ij^+}]$ of each class j^+ of the Dirichlet distribution $D(p^+|\alpha^+)$ from parameter $\alpha^+ = (\alpha_1, \alpha_2, \cdots, \alpha_K, \alpha_u)$ is obtained from the ratio of the sum of all parameters and the parameter of class j^+. In other words,

$$E[p_{ij^+}] = \widehat{p_{ij^+}} = \frac{\alpha_{ij^+}}{\sum_k \alpha_{ik} + \alpha_{iu}} = \frac{\alpha_{ij^+}}{S_i + \alpha_{iu}}. \tag{S1}$$

Here, the symbol $\widehat{p_{ij^+}}$ is used, which is exactly the expected probability value obtained from the Dirichlet distribution output by the m-EDL introduced in Section 2.

Meanwhile, the variance of the Dirichlet distribution $D(p^+|\alpha^+)$ is calculated as follows:

$$Var(p_{ij^+}) = \frac{\alpha_{ij^+} \times \left\{\left(\sum_k \alpha_{ik^+}\right) - \alpha_{ij^+}\right\}}{\left(\sum_{k^+} \alpha_{ik^+}\right)^2 \cdot \left\{\left(\sum_k \alpha_{ik^+}\right) + 1\right\}} = \frac{\alpha_{ij^+} \times \{S_i + \alpha_{iu} - \alpha_{ij^+}\}}{(S_i + \alpha_{iu})^2 \cdot (S_i + \alpha_{iu} + 1)}. \tag{S2}$$

Equation (2) is arranged using equations (S1) and (S2) to determine the form of the likelihood. Substituting equations (S1) and (S2) into equation (2) yields the following:

$$L_i^+(\Theta) = \sum_{j^+} \left\{\left(y_{ij^+} - \frac{\alpha_{ij^+}}{S_i + \alpha_{iu}}\right)^2 + \frac{\alpha_{ij^+} \times (S_i + \alpha_{iu} - \alpha_{ij^+})}{(S_i + \alpha_{iu})^2 \cdot (S_i + \alpha_{iu} + 1)}\right\} \tag{S3}$$

Here, formally writing $S_i^+ = S_i + \alpha_{iu}$ yields the following:

$$L_i^+(\Theta) = \sum_{j^+} \left\{\left(y_{ij^+} - \frac{\alpha_{ij^+}}{S_i^+}\right)^2 + \frac{\alpha_{ij^+} \times (S_i^+ - \alpha_{ij^+})}{S_i^{+2}(S_i^+ + 1)}\right\} = \sum_{j^+} \left\{\left(y_{ij^+} - \widehat{p_{ij^+}}\right)^2 + \frac{\widehat{p_{ij^+}} \cdot (1 - \widehat{p_{ij^+}})}{S_i^+ + 1}\right\} \tag{S4}$$

Furthermore, the final likelihood form is obtained by considering the Kullback–Leibler divergence term, following Sensoy et al.[1].

As in Sensoy et al.'s method[1], I consider the Kullback–Leibler divergence term to regularize the predictive distribution by penalizing the divergences from class u. Transforming equation (S4) ultimately results in the following loss function, which considers all N samples i:

$$L^{+}(\Theta) = \sum_{i=1}^{N} L_i^{+}(\Theta) + \lambda_t \sum_{i=1}^{N} KL\left[D(p_i^{+}|\widetilde{\alpha_i}^{+})||D(p_i^{+}|1)\right]. \quad \text{(S5)}$$

Here, $\widetilde{\alpha_i}^{+} = y_i^{+} + (1 - y_i^{+}) \odot \alpha_i^{+}$ and $\odot$ is the Hadamard product. In addition, λ_t is the annealing coefficient in training epoch *t*.

Differences and correspondence between the EDL and m-EDL outputs

It was stated in Section 2 that for scenarios with K classes, the outputs of EDL and m-EDL are the $(K-1)$- and K-dimensional Dirichlet distributions, respectively. However, it is not the distribution itself but the expected value of the distribution that is used for classification. The expected value of the Dirichlet distribution $D(\boldsymbol{p}|\boldsymbol{\alpha})$ output by EDL is the following for each class $k \in \{1, \cdots, K\}$:

$$\overline{p_k} = \frac{\alpha_k}{\sum_k \alpha_k}. \quad \text{(S6)}$$

By contrast, the expected value of the Dirichlet distribution $D(\boldsymbol{p}^{+}|\boldsymbol{\alpha}^{+})$ output by m-EDL (equation (S1)) is written as follows for each class $k^{+} \in \{1, \cdots, K, \mathrm{u}\}$:

$$\widehat{p_{k^+}} = \frac{\alpha_{k^+}}{\sum_k \alpha_k + \alpha_\mathrm{u}} = \frac{\alpha_{k^+}}{\sum_{k^+} \alpha_{k^+}}. \quad \text{(S7)}$$

In this section, I first describe the difference between these two values. I then describe the relationship between them.

First, what is assumed to be a whole in EDL and m-EDL differs (Fig. S1**a**). Here, the two-class classification of $k \in \{\mathrm{A}, \mathrm{B}\}$, $k^{+} \in \{\mathrm{A}, \mathrm{B}, \mathrm{u}\}$ is taken as an example. It can be seen from equation (S6) that $\overline{p_k}$ is the ratio of each α_k when the position of the blue dashed arrow in Fig. S1**a** is treated as 100%. Meanwhile, $\widehat{p_{k^+}}$ is the ratio of each α_{k^+}, when the position of the solid red arrow in Fig. S1**a** is treated as 100%. This is the reason m-EDL can include class u in addition to K classes and output a probability that sums to 1, as mentioned in Section 2. Moreover, the difference between $\overline{p_k}$ and $\widehat{p_{k^+}}$ is the difference in the extent to which the classes are considered as a whole.

By contrast, Fig. S1**b** shows the correspondence between $\overline{p_k}$ and $\widehat{p_{k^+}}$ using two-class classification $k \in \{\mathrm{A}, \mathrm{B}\}$, $k^{+} \in \{\mathrm{A}, \mathrm{B}, \mathrm{u}\}$ as an example. The plane indicated by the dashed blue lines satisfies $1 = \sum_{k^+=A,\mathrm{B},\,\mathrm{u}} p_{k^+}$, and $\widehat{\boldsymbol{p}_{\boldsymbol{k}^+}}$ is a point on this plane. Moreover, the line indicated by the blue bold dashed line satisfies $1 = \sum_{k=A,B} p_k$, and $\overline{\boldsymbol{p}_{\boldsymbol{k}}}$ is a point on this line. Point $\overline{\boldsymbol{p}_{\boldsymbol{k}}}$ is the projection of $\widehat{\boldsymbol{p}_{\boldsymbol{k}^+}}$ from point $p_\mathrm{u} = 1$ to line $1 = \sum_{k=A,B} p_k$. This is because, from equations (S6) and (S7), the relationship of $\overline{p_\mathrm{A}}:\overline{p_\mathrm{B}} = \widehat{p_\mathrm{A}}:\widehat{p_\mathrm{B}}$ does not break down for $\overline{\boldsymbol{p}_{\boldsymbol{k}}}$ and $\widehat{\boldsymbol{p}_{\boldsymbol{k}^+}}$. From this, it can be seen that the $\widehat{\boldsymbol{p}_{\boldsymbol{k}^+}}$ output by m-EDL also stores the information of $\overline{\boldsymbol{p}_{\boldsymbol{k}}}$, which is the output of the original EDL.

1. Sensoy, M., Kaplan, L. & Kandemir, M. Evidential deep learning to quantify classification uncertainty. *Adv. Neural. Inf. Process. Syst.* **31**; https://proceedings.neurips.cc/paper/2018/hash/a981f2b708044d6fb4a71a1463242520-Abstract.html (2018).

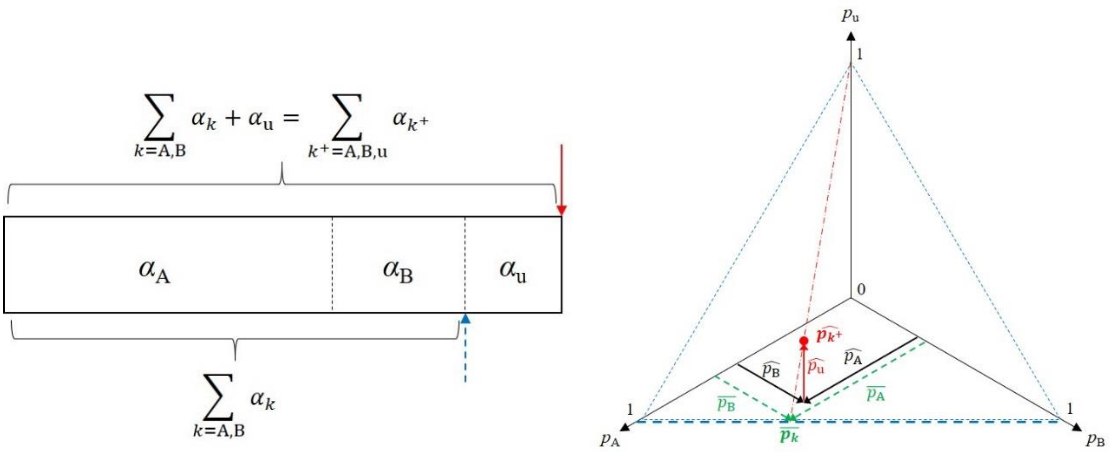

a **b**

Fig. S1 Relationship of $\overline{p_k}$ and $\widehat{p_{k^+}}$. **a** Conceptual diagram of how parameter α is used to obtain $\overline{p_k}$ and $\widehat{p_{k^+}}$ (when $k \in \{A, B\}$, $k^+ \in \{A, B, u\}$). **b** Correspondence between $\overline{p_k}$ and $\widehat{p_{k^+}}$ (when $k \in \{A, B\}$, $k^+ \in \{A, B, u\}$)
